# Nurses Improving Care for Healthsystem Elders – a model for optimising the geriatric nursing practice environment

**DOI:** 10.1111/j.1365-2702.2012.04259.x

**Published:** 2012-11

**Authors:** Elizabeth Capezuti, Marie Boltz, Daniel Cline, Victoria Vaughn Dickson, Marie-Claire Rosenberg, Laura Wagner, Joseph Shuluk, Cindy Nigolian

**Affiliations:** **Authors:***Elizabeth Capezuti*, PhD, RN, FAAN, The Dr John W. Rowe Professor in Successful Aging, New York University College of Nursing, New York, NY; *Marie Boltz*, PhD, RN, CRNP, NHA, John A. Hartford Foundation Claire M. Fagin Fellow & Assistant Professor, New York University College of Nursing, New York, NY; *Daniel Cline*, MSN, RN, CRNP, John A. Hartford Foundation Building Academic Geriatric Nursing Capacity Scholar & PhD Candidate, New York University College of Nursing, New York, NY; *Victoria Vaughn Dickson*, PhD, RN, Assistant Professor, New York University College of Nursing, New York, NY; *Marie-Claire Rosenberg*, PhD, RN, Assistant Professor, New York University College of Nursing, New York, NY; *Laura Wagner*, PhD, RN, Assistant Professor, New York University College of Nursing, New York, NY; *Joseph Shuluk*, BA, Assistant Research Scientist, New York University College of Nursing, New York, NY; *Cindy Nigolian*, MS, RN, GCNS, BC, NICHE Clinical Administrator, New York University College of Nursing, New York, NY, USA

**Keywords:** elderly people, geriatric care environment, geriatrics, healthcare organisations and systems, hospitals, nurse practice environment, nursing, patient care, quality, quality of geriatric care

## Abstract

**Aims and objectives:**

To explain the relationship between a positive nurse practice environment (NPE) and implementation of evidence-based practices. To describe the components of NICHE (Nurses Improving Care for Healthsystem Elders) programmes that contribute to a positive geriatric nursing practice environment.

**Background:**

The NPE is a system-level intervention for promoting quality and patient safety; however, there are population-specific factors that influence the nurses’ perception of their practice and its’ relationship with patient outcomes. Favourable perceptions of the geriatric-specific NPE are associated with better perceptions of geriatric care quality.

**Designs:**

Discursive paper.

**Method:**

In this selective critical analysis of the descriptive and empirical literature, we present the implementation of geriatric models in relation to the NPE and components of the NICHE programme that support hospitals’ systemic capacity to effectively integrate and sustain evidence-based geriatric knowledge into practice.

**Results:**

Although there are several geriatric models and chronic care models available, NICHE has been the most successful in recruiting hospital membership as well as contributing to the depth of geriatric hospital programming.

**Conclusions:**

Although all geriatric care models require significant nursing input, only NICHE focuses on the nursing staff’s perception of the care environment for geriatric practice. Studies in NICHE hospitals demonstrate that quality geriatric care requires a NPE in which the structure and processes of hospital services focus on specific patient care needs.

**Relevance to clinical practice:**

The implementation of evidence-based models addressing the unique needs of hospitalised older adults requires programmes such as NICHE that serve as technical resources centre and a catalyst for networking among facilities committed to quality geriatric care. Unprecedented international growth in the ageing population compels us to examine how to adapt the successful components of NICHE to the distinctive needs of health systems throughout the world that serve older adults.

## Background

It is well established that the nurse practice environment (NPE) is essential to nurse satisfaction and subsequently to nurse-sensitive patient outcomes. Studies exploring the NPE in acute care settings serving specific patient populations such as persons receiving care in AIDS, oncology and critical care units suggest care models focused on population-specific factors have a positive impact on nurses’ perceptions of their practice environments (Aiken *et al.* 1999, Choi *et al.* 2004, Friese *et al.* 2008). Further, units with specialised care models focused on older adults, such as Acute Care of the Elderly (ACE) units, have demonstrated better outcomes than units that do not have specialised care models (Counsell *et al.* 2000).

Older adults currently use a disproportionately higher amount of health services compared with other age groups (Hall *et al.* 2010) and are more likely to experience complications (e.g. delirium, functional decline, pressure ulcers) and adverse events (e.g. fall-related injuries) during hospitalisation (Boltz *et al.* 2010a,b,c,d,e,f, Kim *et al.* 2010). As the older adult population grows, there will be a significant increase in the demand for health services. The number of older hospitalised patients makes it impractical to consider segregating older adults on specific units; instead, most units in a hospital should adopt ageing-sensitive principles (Capezuti & Brush 2009, Malone *et al.* 2010). This implies embedding such principles at the hospital level to ensure that the older adult receives high-quality care throughout their hospital experience, regardless of unit location. The NICHE programme facilitates the integration of geriatric care models at the hospital and health system levels to ensure older adults receive the specialised care they need no matter where they are in the hospital or health system. This paper aims to (1) explain the relationship between a positive NPE and implementation of evidence-based practices and (2) to describe the components of the NICHE (Nurses Improving Care for HealthSystem Elders) programmes that contribute to a positive geriatric nursing practice environment.

## Design

This discursive paper presents a selective critical analysis of the descriptive and empirical literature addressing the implementation of geriatric models in relation to the NPE.

## Method

This study was performed as a discursive paper that included a brief description of the NPE and the evidence to support population-specific factors that influence the nurses’ perception of their practice and its relationship with patient outcomes. A summary of the major geriatric practice models is presented; although all models require significant nursing input, only NICHE promotes a significant role for nursing to address geriatric hospital practice. Finally, this review concludes with a synopsis of the components of the NICHE programmes that support the geriatric nursing practice environment by assisting hospitals’ systemic capacity to effectively integrate and sustain evidence-based geriatric knowledge into practice.

### Nurse practice environment

The general NPE is described as the organisational characteristics of a work setting that facilitate or constrain professional nursing practice (Lake 2002). These characteristics include the quality of nurses’ relationships with managers and physicians and the status of nurses within the hospital structure. Nurses’ dissatisfaction with their work environment has been linked to lack of job-related empowerment and engagement such as perceived lack of autonomy, limited supervisor support and few rewards for job performance (Aiken *et al.* 2002a, Needleman *et al.* 2002, Kovner *et al.* 2006). Current evidence indicates the significant influence of the NPE, at both the hospital and unit levels, on nurse, patient and organisational outcomes (Aiken *et al.* 2002a,b, Kovner *et al.* 2007, Aiken *et al*. 2008, Friese *et al.* 2008). Thus, models that promote a positive NPE have become regarded as a potential system-level intervention for promoting quality and patient safety (Kim *et al.* 2009).

Organisational interventions to improve the professional practice environment include supporting nurses to perform at the highest level of practice, to function successfully within an interdisciplinary team and to mobilise resources quickly (Friese *et al.* 2008). Additionally, nurses’ perceptions of quality care have identified teamwork, access to resources, time constraints and technologies that support communication and documentation as important features to the successful delivery of quality patient care (Cline *et al.* 2011, D. Cline, E. Capezuti, V. Dickson & C. Kovner, New York University College of Nursing, New York, NY, unpublished PhD Thesis). It is not clear, however, whether these factors are effective in all settings. For example, does the general NPE capture aspects of the nurses’ work specific to delivering high-quality care to hospitalised older adults or does it need to be specialised?

Studies describing the NPE in acute care units serving specific patient populations (AIDS, oncology and critical care units) imply that that there are other dimensions that need to be specialised to the population and that influence the nurses’ perception of their practice as well as its relationship with patient outcomes (Aiken *et al.* 1999, Choi *et al.* 2004, Friese *et al.* 2008). Testing of geriatric acute care models has shown that complications common to hospitalised older adults can be prevented. Therefore, is it possible that a hospital’s ability to embed geriatric principles and strengthen the geriatric expertise of providers can yield improvements in nurse, patient and organisational outcomes?

Kim *et al.* (2009) used the valid and reliable Practice Environment Scale of the Nursing Work Index to measure the general NPE and subscales of the Geriatric Institutional Assessment Profile (GIAP) (Abraham *et al.* 1999, Kim *et al.* 2007, Boltz & Capezuti 2008, Boltz *et al.* 2009, 2010a,b,c,d,e,f) to quantify the geriatric-specific nursing practice environment in a sample of 192 registered nurses who worked at three non-profit acute care hospitals located in two metropolitan areas in New York State. Kim *et al.* (2007) examined the relationship of the NPE to nurse-perceived quality of geriatric care in hospitals using both general (Lake 2002) and geriatric-specific (Boltz *et al.* 2008b) NPE scales, while adjusting for hospital and nurse covariates. Nurse-perceived quality of geriatric care was measured by the 10-item Aging-Sensitive Care Delivery (ASCD) Scale of the GIAP (Kim *et al.* 2007). The ASCD Scale quantifies nurses’ perceptions of the extent to which hospitals provide evidence-based care that focuses on the unique needs, situations and preferences of older hospitalised adults (Boltz *et al.* 2008a,b). The general NPE was negatively related to nurse-perceived quality of geriatric care, but total geriatric-specific NPE was positively related to nurse-perceived quality of geriatric care after adjusting for nurse and hospital covariates. Consistent with previous studies (Boltz *et al.* 2008a,b, McKenzie *et al.* 2011), these findings suggest that more favourable perceptions of the geriatric-specific NPE are associated with better perceptions of geriatric care quality (Kim *et al.* 2009). Quality geriatric care may require a focus on the structures and processes of the NPE specific to a populations’ care needs, such as older adults, rather than only focusing on supporting nurses’ more generally in their professional NPE. Structure and processes of hospital nursing services also need to focus on specific patient care needs. Organisational support should address nurse competencies related to the complex interdisciplinary care management of older adults and the resources needed to improve the safety and outcomes of hospitalised older adults (Kim *et al.* 2007, 2009, Boltz *et al.* 2008b, McKenzie *et al.* 2011).

### Geriatric models of care

Over the past three decades, numerous geriatric care models emerged; these include the Geriatric Consultation Service, the ACE (Acute Care for the Elderly) unit, the NICHE (Nurses Improving Care for Healthsystem Elders) initiative, the Geriatric Resource Nurse (GRN) model and the HELP (Hospital Elder Life Program). In general, these models target the prevention of complications that occur more commonly in older adults and the hospital factors that contribute to complications by employing evidence-based, ageing-sensitive interventions, promoting interdisciplinary communication and emphasising discharge planning (Steele 2010). These programmes provide ample evidence to demonstrate positive patient and institutional outcomes related to all or most of these objectives (Capezuti & Brush 2009). Despite the fact that these models are empirically driven and clinically successful, hospitals are reluctant to adopt them (Jayadevappa *et al.* 2003, SteelFischer *et al.* 2011). The most frequently implemented model is NICHE. We assert that NICHE’s focus on improving outcomes by positively influencing the geriatric nursing practice environment is the key to its success. Although geriatric models of care differ in their approach, all require significant nursing input; however, only NICHE aligns its approach to nurse involvement in hospital decision-making regarding care of older adults. NICHE principles and resources are congruent with professional nursing practice models (Kim *et al.* 2009, Boltz *et al.* 2010a,b,c,d,e,f), and NICHE hospitals are also more likely to have Magnet designation (Nickoley 2010, Rosenberg *et al.* 2010).

### Nurses Improving Care for Healthsystem Elders

Nurses Improving Care for Healthsystem Elders (NICHE) provides the necessary resources and technical support to assist hospitals’ systemic capacity to effectively embed evidence-based geriatric knowledge into practice. NICHE is evidence-based programme that has been evolving through research conducted over the last 20 years. The core components of a system-wide, acute care programme designed to meet the needs of older adults are grouped into eight categories (guiding principles, leadership, organisational structures, the physical environment, patient- and family-centred approaches, ageing-sensitive practices, geriatric staff competence, and interdisciplinary resources and processes) (Boltz *et al.* 2010a,b,c,d e,f). Each category is viewed as an important element and, when combined, represents a unified system-wide approach to improving geriatric acute care. These elements, in addition to associated resources, programmes and activities, provide a framework for a hospital to use in the planning, implementation and evaluation of an acute care model for older adults. This framework also guides the international NICHE programme.

Nurses Improving Care for Healthsystem Elders is a programme of the Hartford Institute for Geriatric Nursing at New York University College of Nursing. NICHE functions similar to a not-for-profit professional membership organisation in that health facilities pay an initiation and annual membership fee; however, it is also a professional collaborative as membership requires organisational commitment including participation of senior personnel in a NICHE leadership training programme and ongoing demonstration of active geriatric programming. Owing to this high level of engagement, most NICHE sites are resource-rich institutions. Currently, NICHE is comprised of approximately 330 NICHE active member sites, representing individual hospitals and almost 100 health systems in the United States and Canada. NICHE coordinators at each site guide the core steering committee to implement and sustain the NICHE programme such as influencing the incorporation of geriatric-specific protocols and staff education programmes. Coordinators and/or advanced practice nurses provide direct clinical care of patients, support interdisciplinary collaboration and mentor staff nurses to function as peer consultants to other staff (Capezuti *et al.* 2010).

Nurses Improving Care for Healthsystem Elders site designation is determined by both internal and external indicators. Internally, each site must demonstrate administrative commitment, active geriatric programming, defined NICHE leadership, active provider involvement and an action plan that includes clear measurable outcomes for evaluation. Externally, representatives from NICHE sites participate in the NICHE network by attendance and presentation at national conferences, webinars and other activities that contribute to NICHE resource sharing and development.

The national office at New York University College of Nursing supports a centralised, web-based portal for educational, clinical and operational tools as well as the web-based survey, the GIAP. The general pages of the NICHE website (http://www.nicheprogram.org), along with *Evidence-Based Geriatric Nursing Protocols for Best Practice* (Boltz *et al.* 2012), serve the wider community of hospitals. The NICHE ‘Members Only’ web pages include extensive resources relevant for clinical practice, organisational strategies, staff education and training. Members access the ‘Members Only’ section of the NICHE website to participate in audio/webcast conferences, use an extensive learning management system and dialogue among NICHE members via discussion forums (Capezuti *et al.* 2010). Additionally, sites administer the GIAP to assess their institutional readiness to provide quality elder care prior to implementing NICHE.

### NICHE collaborative approach

Collaboration is emphasised at the unit, interdisciplinary, organisational and the NICHE membership levels.

#### Unit level: GRN model

At the NICHE unit level, the implementation of the GRN model fosters the integration of evidence-based geriatrics within clinical practice. This educational and clinical intervention model prepares staff nurses as clinical resource leaders on geriatric issues to other nursing staff on their unit and supports their active role on the interdisciplinary team (Fulmer 2001, Lee & Fletcher 2002, Lopez *et al.* 2002, Pfaff 2002, Mezey *et al.* 2004). The GRN model provides staff nurses, via education and modelling by a geriatric advanced practice nurse, with explicit content to identify and address specific geriatric syndromes, such as falls and confusion, and to implement care strategies that discourage the use of restrictive devices and promote patient mobility (Boltz *et al.* 2010a,b,c,d,e,f). GRNs function in all types of units that serve older adults, including specialty units, critical care areas (Boltz *et al.* 2010, Mullin *et al.* 2011) and dementia programmes (Guthrie *et al.* 2006, Allen & Close 2010).

The GRN model is considered the foundation for improving geriatric care. The underlying goal of the GRN model is to improve the geriatric knowledge and expertise of the bedside nurse, which is essential to implementing system-wide improvement in the care of older adult patients. Nurses are supported by a NICHE coordinator and in those hospitals with the resources, a geriatric advanced practice nurse, geriatrician and other geriatric health specialists. The goal is to promote nurses as leaders who use patient rounds, bedside teaching, geriatric interest groups, hospital committees and geriatric initiatives to increase geriatric knowledge and maximise the coordination of care across disciplines (Fletcher *et al.* 2007, Capezuti *et al.* 2010). GRNs are actively engaged in educating nursing assistants as geriatric patient care associates (Weitzel & Robinson 2004).

#### Interdisciplinary collaboration

The care of older adults requires an interdisciplinary approach. NICHE is a nurse-led programme that is similar to other geriatric programmes and models in which nursing plays a crucial role in coordinating the interdisciplinary team members’ contributions as well as operationalising the programme goals within the institution. The NICHE coordinator at each site functions in a leadership role (facilitating a hospital-wide steering committee, teaching and mentoring others and changing systems of care) to promote a geriatric-responsive practice environment for all disciplines. This is accomplished by educating all team members through a 5-part NICHE Introduction to Gerontology learning programme that is designed to increase the hospital personnel’s sensitivity to the ageing process by improving the recognition of age-related changes in older adult patients and enhancing communication skills with older patients and their families. This introductory series provides a foundation for developing geriatric sensitive care across all hospital departments (Stevenson *et al.* 2009). A second feature, evidence-based clinical protocols, provides the structured interdisciplinary processes for an effective application of a team approach to common geriatric issues such as pressure ulcers, catheter-associated urinary tract infections (CAUTIs), delirium and falls (Capezuti *et al.* 2008). Additionally, NICHE organisational strategies, which are authored by clinicians (e.g. nurses, pharmacists) and administrators/managers, provide project management tools to embed evidence-based care in daily operations.

#### Institutional-level support

Without institutional-level support, unit-level interdisciplinary and individual clinician efforts are unlikely to be sustained. Successful NICHE hospitals evaluate a shared vision for geriatric care within their hospital’s mission, assess organisational readiness for NICHE, build support at the hospital and community levels, propose a business case for sustainability and develop an action plan for implementation (Bond *et al.* 2010, Wurmser 2010). Hospitals using this approach are more likely to improve the experience and outcomes of hospitalisation for older adults (Boltz *et al.* 2008a). Some NICHE hospitals have entered into partnerships with schools of nursing to educate frontline staff and support the implementation and evaluation of geriatric quality initiatives (Hendrix *et al.* 2011).

#### Site-level collaboration

A key contribution to sustained NICHE site involvement is their mutual use of, and contribution to, collaborative venues facilitated by NICHE. These include discussion forums for NICHE sites whereby NICHE coordinators, site staff and administrators’ dialogue about common issues they face in promoting quality geriatric care. Webinars and the NICHE Solutions series (http://nicheprogram.org/join-solutions) provide audio and web-based examples of successful NICHE site programmes that can be adapted for use in other sites. Finally, an annual conference provides another venue for sites to describe and disseminate their innovative approaches and measurable outcomes through poster presentations and oral paper presentations. NICHE resources are developed based on successful, evidence-based examples from NICHE sites as well as in response to NICHE site requests.

#### Contributing to research

In addition to their role of translating research to the bedside, NICHE sites can also participate in a network of study sites for research. Sites can initiate research projects that they are interested in implementing in multiple sites, while researchers can use the NICHE network to recruit sites through the NICHE Research Team at NYU. The latter is also responsible for the analyses of de-identified benchmarking data from the GIAP (Kim *et al.* 2007, 2009, Boltz *et al.* 2008b, McKenzie *et al.* 2011). NICHE sites thus contribute to a growing body of knowledge of effective mechanisms to improve care processes (Boltz *et al.* 2010a,b,c,d,e, 2011, in press, Kim *et al.* 2010, Wald *et al.* 2010) and to implement geriatric programming.

### NICHE outcomes

Improved geriatric outcomes at the nurse, patient and organisational levels include both single-site and multisite studies of NICHE hospitals. Units that have implemented the GRN model have demonstrated significantly improved nurse knowledge and attitudes related to incontinence (Pfaff 2002), improved nurse knowledge related to pressure ulcers, restraint use, incontinence and sleep (Fitzpatrick *et al.* 2004) and more positive attitudes about pressure ulcer and restraint management (Fitzpatrick *et al.* 2004). Among clinical process measures relevant to geriatric practice, NICHE units have been associated with reduced physical restraint use (Pfaff 2002, Swauger & Tomlin 2002, Turner *et al.* 2001, Fitzpatrick *et al.* 2004), increased compliance with incontinence protocol (Pfaff 2002) and improved documentation and family support (Fitzpatrick *et al.* 2004).

Decreased incidence of patient complications include new onset of confusion or delirium (Guthrie *et al.* 2002, 2006, Lee & Fletcher 2002, Swauger & Tomlin 2002), facility-acquired incontinence (Turner *et al.* 2001), urinary tract infection rate (Swauger & Tomlin 2002), pneumonia (Swauger & Tomlin 2002), mobility loss (Turner *et al.* 2001), pressure ulcers (Swauger & Tomlin 2002), reported pain (Turner *et al.* 2001) and fall-related injuries (Swauger & Tomlin 2002). Reported improved organisational outcomes are decreased length of stay (Turner *et al.* 2001, Swauger & Tomlin 2002) and reduced costs of care (Fulmer *et al.* 2002, Guthrie *et al.* 2002, Pfaff 2002, Swauger & Tomlin 2002).

The GIAP is a psychometrically validated instrument used by NICHE hospitals to evaluate staff knowledge as well as perceptions of the care environment specific to care of older adults and the quality of geriatric care delivered (Fulmer *et al.* 2002, Kim *et al.* 2007, Boltz *et al.* 2009). Eight acute care American hospitals administered the GIAP to direct care nurses prior to (*n* = 821) and following (*n* = 942) NICHE implementation (Boltz *et al.* 2008a,b). Controlling for hospital and nurse characteristics, both nurse perceptions of the geriatric nursing practice environment (*p*< 0·0001) and quality of geriatric care (*p* = 0·0004) increased following NICHE implementation. These study findings indicate that organisational support for geriatric nursing exerts an important influence upon perceptions of quality of geriatric care (Boltz *et al.* 2008a,b). Although these outcomes are encouraging, this study did not use direct measures of patient outcomes. Currently, Dr Marie-Claire Rosenberg at NYU College of Nursing is evaluating three years of Medicare claims data of older patients hospitalised for a hip fracture or acute myocardial infarction to compare NICHE with non-NICHE hospitals on both nurse-sensitive patient (pressure ulcers, postoperative infection, failure-to-rescue) and organisational (in-hospital mortality and length of stay) outcomes.

### NICHE programmatic development and research

Nurses Improving Care for Healthsystem Elders sites influence the development of elder-responsive programmes and models. For example, NICHE health systems that include rural hospitals have had difficulty broadening their geriatric initiatives to these sites because their location or size precludes employing geriatric specialists. Although NICHE resources can all be accessed using a web-based portal on the NICHE website and implementation is supported by experienced regional mentors, there have been few NICHE hospitals located in rural counties. Based on a model used by geriatricians in NICHE sites in the Aurora Health System (Malone *et al.* 2010), NICHE is developing and testing a model of an ‘e-GAPN’ (Geriatric Advanced Practice Nursing) with 20 small, rural hospitals in northern New York State. We are exploring how an e-GAPN can promote the geriatric expertise of direct care nurses as well as mentor hospital administrators and unit managers within an online learning community. Long-distance communication strategies (e.g. discussion forums, webinars, tele/video conferencing) can potentially expand access to geriatric specialists while improving the core geriatric competences of direct care clinicians (Capezuti 2010). Additionally, another NYU study is using qualitative and quantitative methods to explore rural nurses’ perceptions of their work environment to identity processes of care important to the delivery of quality geriatric care in rural hospitals (Cline *et al.* 2011, D. Cline, E. Capezuti, V. Dickson & C. Kovner, New York University College of Nursing, New York, NY, unpublished PhD Thesis).

Nurses Improving Care for Healthsystem Elders is also working with researchers to improve processes of care. Dr Marie Boltz of NYU and Dr Barbara Resnick of the University of Maryland are testing the feasibility of a system-level intervention designed to optimise functional performance throughout the hospital stay and facilitate carryover of that care to the postacute setting (e.g. home, rehabilitation site). They aim to improve patient outcomes, specifically physical function, physical activity, self-efficacy and outcome expectations for function, while lowering the incidence of adverse events (falls, pressure ulcers, infections) at discharge (Boltz *et al.* 2010a,b,c,d,e,f, in press).

Dr Heidi Wald of the University of Colorado is conducting a study of 20 NICHE hospitals with the following goals: (1) dissemination of an electronic method for tracking CAUTIs and catheter duration and (2) determination of the effect of the feedback of these data on processes of care (catheter duration) and outcomes (CAUTIs) (http://www.ucdenver.edu/academics/colleges/medicalschool/departments/medicine/hcpr/cauti/Pages/default.aspx). The CAUTI intervention consists of a) electronic audit and feedback reports to hospitals on unit-specific, patient-level urinary catheter duration and CAUTI rates and b) an educational session on CAUTI prevention and evidence-based best practices for urinary catheter management (Wald *et al.* 2010).

Finally, NICHE is collaborating with other professional organisations to develop and test the effectiveness of new resources to improve the patient and family experience of hospitalisation. NICHE, in partnership with the American Association of Retired Persons (AARP) and the American Journal of Nursing, will create educational materials and toolkits to help nurses and social workers support family caregivers with a particular focus on the needs of diverse communities. These family-centred practices will be evaluated in representative NICHE hospitals prior to national dissemination.

## Conclusions

Quality geriatric care requires a NPE in which the structure and processes of hospital services focus on specific patient care needs. Although there are several geriatric models and chronic care models available, NICHE has been the most successful in recruiting hospital membership as well as contributing to the depth of geriatric hospital programming. All geriatric models of care include a high level of nursing input but only NICHE stresses nurse involvement in hospital decision-making regarding care of older adults. This professional nursing practice perspective support nurse competencies related to the complex interdisciplinary care management of older adults and the resources they need to improve the safety and outcomes of hospitalised older adults. Clearly, the ageing of our worldwide population requires a systematic incorporation of evidence-based geriatric principles in healthcare settings serving older adults.

## Relevance to clinical practice

The implementation of evidence-based interventions and models addressing the unique needs of hospitalised older adults requires programmes such as NICHE that serve as technical resources centre and a catalyst for networking among facilities committed to quality geriatric care. Unprecedented growth in the ageing population worldwide compels us to examine how to adapt the successful components of NICHE to the distinctive needs of health systems beyond North America. This means much more than simply translating NICHE resources. Modifications in other parts of the world will need to take into account the financing and organisation of health care, the role of nurses and geriatric specialists (across disciplines) as well the social and economic issues that older adults face, which impacts health status.
